# Public Access and Use of Health Research: An Exploratory Study of the National Institutes of Health (NIH) Public Access Policy Using Interviews and Surveys of Health Personnel

**DOI:** 10.2196/jmir.1827

**Published:** 2011-11-21

**Authors:** Jamie O'Keeffe, John Willinsky, Lauren Maggio

**Affiliations:** ^1^Stanford University School of EducationStanford, CAUnited States; ^2^Lane Medical LibraryStanford University School of MedicineStanford, CAUnited States

**Keywords:** Health policy, evidence-based practice, information storage and retrieval, access to information, information literacy, health personnel

## Abstract

**Background:**

In 2008, the National Institutes of Health (NIH) Public Access Policy mandated open access for publications resulting from NIH funding (following a 12-month embargo). The large increase in access to research that will take place in the years to come has potential implications for evidence-based practice (EBP) and lifelong learning for health personnel.

**Objective:**

This study assesses health personnel’s current use of research to establish whether grounds exist for expecting, preparing for, and further measuring the impact of the NIH Public Access Policy on health care quality and outcomes in light of time constraints and existing information resources.

**Methods:**

In all, 14 interviews and 90 surveys of health personnel were conducted at a community-based clinic and an independent teaching hospital in 2010. Health personnel were asked about the research sources they consulted and the frequency with which they consulted these sources, as well as motivation and search strategies used to locate articles, perceived level of access to research, and knowledge of the NIH Public Access Policy.

**Results:**

In terms of current access to health information, 65% (57/88) of the health personnel reported being satisfied, while 32% (28/88) reported feeling underserved. Among the sources health personnel reported that they relied upon and consulted weekly, 83% (73/88) reported turning to colleagues, 77% (67/87) reported using synthesized information resources (eg, UpToDate and Cochrane Systematic Reviews), while 32% (28/88) reported that they consulted primary research literature. The dominant resources health personnel consulted when actively searching for health information were Google and Wikipedia*,* while 27% (24/89) reported using PubMed weekly. The most prevalent reason given for accessing research on a weekly basis, reported by 35% (31/88) of survey respondents, was to help a specific patient, while 31% (26/84) were motivated by general interest in research.

**Conclusions:**

The results provide grounds for expecting the NIH Public Access Policy to have a positive impact on EBP and health care more generally given that between a quarter and a third of participants in this study (1) frequently accessed research literature, (2) expressed an interest in having greater access, and (3) were aware of the policy and expect it to have an impact on their accessing research literature in the future. Results also indicate the value of promoting a greater awareness of the NIH policy, providing training and education in the location and use of the literature, and continuing improvements in the organization of biomedical research for health personnel use.

## Introduction

Funded research should be accessible and open without cost (or with very minimal cost) to all providers…[since charging] such high rates for access to crucial information is detrimental to health care.

The above quote was submitted by a health care provider in response to our recent survey on awareness of the 2008 National Institutes of Health (NIH) Public Access Policy [1]. The policy requires all research publications resulting from NIH funding to be made publicly accessible through deposit in the National Library of Medicine’s PubMed Central within 12 months of publication. This NIH policy is expected to provide public access to some 80,000 biomedical research articles annually [2] and represents a broader trend within scholarly communication toward “open access” [3]. For example, a number of funding agencies, journals, and institutions, such as the Wellcome Trust, *Journal of Medical Internet Research*, and Stanford University, have adopted open access policies. It is imperative that these policies be assessed for their (potential) impact and ability to guide future policies.

Health personnel’s access to research literature has taken on greater cogency with the ongoing emphasis on evidence-based practice (EBP) in health care. EBP is the “conscientious, explicit, and judicious use of current best evidence in making decisions about the care of individual patients” [4]. *Best evidence* is defined as the current evidence from the research literature [4,5]. However, research on information seeking among health personnel has repeatedly demonstrated their bias toward easily accessible information [6-8]. For example, in a study of 47 physicians during a half-day of practice, Covell [9] found that physicians raised a total of 269 questions about patient management. Roughly two-thirds of these questions were not pursued, a finding that has been repeated by Gorman and Helfand [10]. Of the 30% of questions that were pursued, the physicians most frequently sought answers from other health professionals [9].

Other studies, such as Haug’s [11] meta-review of 12 physician studies, found that physicians prefer to consult local and easily accessible information sources, such as colleagues or local textbooks. Although nurses have been less frequently studied, they seem to have similar preferences for local, easily accessible information sources [6]. Slawson and Shaughnessy [8] call this phenomenon “satisficing,” in which health personnel are “satisfied with the information they have at hand, sacrificing quality for convenience.” Instead of seeking the best evidence, many providers rely on summaries and guidelines, whether or not they are evidence-based [8].

These findings raise important questions about health personnel’s level of access to “best evidence” from the research literature. Currently, the leading sources of easily accessible evidence are point-of-care (POC) services, such as UpToDate, ClinicalEvidence, and DynaMEd, among others, which generally provide synthesized accounts of evidence on major medical topics. In the study reported here, all participants had access through their institution to UpToDate, which is self-described as a clinical decision support system based on current evidence [12]. While the availability of such resources raises questions about the added value that increased access to the research literature provides, studies have demonstrated the limitations of preappraised sources in answering complex clinical questions when compared with the research literature [13,14]. While health personnel have been found to prefer POC resources to PubMed for primary literature [15], POC resources have been questioned on the level [16,17] and currency [18] of evidence that they utilize in creating their syntheses of medical topics [16].

This study measured current research usage among health personnel from a hospital and community clinic in order to assess the potential impact of the NIH Public Access Policy as well as provide a preliminary set of measures for future policy assessments. These measures include (1) perceived quality of current access to information, (2) source and frequency of access, (3) search strategies, (4) reasons for access, and (5) awareness of the NIH Public Access Policy.

## Methods

### Population

The Stanford University Institutional Review Board reviewed and approved the study in June 2010. The study was performed at two health care facilities in Northern California, an independent teaching hospital and a community clinic, from July 2010 through November 2010. The teaching hospital has fewer than 200 beds. The community clinic includes multiple clinical sites involving fewer than 100 physicians and nurses. In 2009, the vast majority of the clinic’s patients were at or below the federal poverty level, and almost all were either uninsured or on public health insurance (eg, Medi-Cal).

### Interviews

During July and August of 2010, 14 interviews were conducted. Interview participants were recruited via each institution’s email listserv. Of those interviewed, 14 agreed to participate, 4 of whom also completed an optional PubMed interview and training. In all, 7 participants were hospital employees and 3 were clinic employees; 6 women and 4 men were interviewed ranging in age from 30 to 50 years. The participants included 5 physicians, 4 nurses, and 1 neuropsychologist; 2 of the physicians also held administrative roles. Interview prompts focused on participant work roles, tasks, characteristics of information needs, sources of information, and outcomes. Questions were directed toward information use, in general (eg, “What resources do you use to get the information you need?”), and use of research, in particular (eg, “Do you ever consult research articles in journals, either in print or online?”)

### Surveys

The survey was informed by previous studies [19] and the preliminary results of the interviews. Survey respondents were recruited via each institution’s email listserv. Members of each institution’s listserv were sent a recruitment email that contained a link to the online survey and was open to any participant that accessed the link. The survey itself and the ten questions within it were voluntary. The online survey contained a total of five screens: the first screen included background information about the study, screens two through four each contained two questions, and screen five contained four questions.

Survey respondents included 90 health personnel across the two sites, 88 of which completed the survey. Of the survey respondents, 32% (28/88) were from the clinic, representing a 32% response rate for clinic personnel. A total of 68% (60/88) of the respondents were from the hospital; however, a response rate for this site could not be determined as the number of health personnel on the email listserv was unknown. Of the respondents, 79% (69/87) were female and 21% (18/87) were male. In addition, 46% (40/88) were physicians, 26% (23/88) were nurses, and 28% (25/88) were other health personnel, such as physician assistants, psychologists, and social workers.

## Results

### Quality of Access to Health Information

Survey respondents were asked to characterize their current level of access to research articles as excellent, good, poor, very poor, or not applicable. Of the survey respondents, 65% (57/88) reported their level of access to be good or excellent, while 32% (28/88) respondents reported access to be poor or very poor (see Table 1). A physician at the hospital summarized the issue of a lack of access:

H-P10 (Bracketed numbers refer to interview participants) I’ve looked [online] at some abstracts for articles that I couldn’t get. I’ve thought, ‘Wow, that’s a really good article. It’s too bad I can’t read it’.

**Table 1:** Perceived quality of access

	n (%) (Total N = 88)
Excellent: I have access to all the research articles that I need.	8 (9%)
Good: I have access to most of the research articles that I need.	49 (56%)
Poor: I frequently have difficulty getting the research articles that I need.	26 (30%)
Very poor: I always have great difficulty getting the research articles I need.	2 (2%)
Not applicable: I do not need access to research articles.	3 (3%)

### Source and Frequency of Access

Survey respondents were asked how often they consult different types of information sources (see Table 2). UpToDate was identified apart from other POC services (such as MDConsult) because it alone was universally available to all participants. In that light, the results for UpToDate, as a POC, can be combined with the category *other POC* services, suggesting that 77% (67/87) of participants used a POC on a weekly basis.

**Table 2:** Source and frequency of access

	Weekly n (%)	Monthly n (%)	Less Frequently n (%)	Totals N (%)
Other medical professionals	73 (83%)	7 (8%)	8 (9%)	88 (100%)
Reference books or websites	59 (69%)	18 (21%)	10 (10%)	86 (100%)
Clinical guidelines/protocols	41 (47%)	26 (30%)	21 (23%)	88 (100%)
UpToDate	39 (45%)	4 (5%)	44 (50%)	87 (100%)
Review articles	34 (39%)	30 (34%)	24 (27%)	88 (100%)
Original research articles	28 (32%)	29 (33%)	29 (35%)	88 (100%)
Other POCs (eg, MDConsult)	28 (32%)	13 (15%)	46 (53%)	87 (100%)
Personal journal subscriptions	21 (24%)	19 (22%)	48 (54%)	88 (100%)

### Search Strategies

Respondents and interviewees used a number of strategies to find health information outside of POC resources. In all, 67% (57/85) of respondents reported weekly use of Google or Wikipedia (as a guide to related sources), and 27% (24/89) reported weekly use of PubMed or MEDLINE (see Table 3). Only 9% (8/87) of survey respondents reported weekly access to an online university library collection.

**Table 3:** Search strategy

	Weekly n (%)	Monthly n (%)	Less Frequently n (%)	Totals N (%)
Google or Wikipedia	57 (67%)	18 (21%)	10 (12%)	85 (100%)
PubMed or MEDLINE	24 (27%)	21 (24%)	44 (50%)	89 (100%)
Remote access to university library	8 (9%)	1 (1%)	78 (90%)	87 (100%)

### Reasons for Access

In all, eight themes emerged from our interview data related to reasons for accessing research (see Table 4). Of those eight, the three most frequently reported reasons for consulting research on a weekly basis were “informing my understanding of a specific patient” (35%, 31/88), “out of general interest” (31%, 26/88), and “informing and updating clinical practice, in general” (25%, 22/88).

**Table 4:** Reasons for accessing health research

	Weekly n (%)	Monthly n (%)	Less Frequently n (%)	Totals N (%)
With regard to a specific patient	31 (35%)	23 (26%)	34 (39%)	88 (100%)
Out of general interest	26 (31%)	18 (21%)	40 (48%)	84 (100%)
Informing clinical practice in general	22 (25%)	29 (33%)	37 (42%)	88 (100%)
Educating patients and their families	15 (17%)	25 (29%)	47 (54%)	87 (100%)
Training or informing health personnel	16 (18%)	17 (19%)	55 (63%)	88 (100%)
Preparing for school or licensure	11 (13%)	7 (8%)	70 (89%)	88 (100%)
Preparing for presentation or teaching	7 (8%)	16 (18%)	65 (74%)	87 (100%)
Writing protocols, articles, or books	3 (3%)	8 (9%)	77 (88%)	88 (100%)

### Policy Awareness

Survey respondents were asked whether they were previously aware of the NIH Public Access Policy, and 27% (23/86) of participants reported being aware, while 73% (63/86) reported they were not familiar with the policy. The survey respondents reporting awareness of the policy also indicated more frequently utilizing research (65% on a weekly basis) than respondents reporting no policy awareness. When asked whether free online access enabled by the policy is likely to have an impact on the frequency of their accessing research articles, 83% (72/87) of respondents reported that some impact is likely, 12% (10/87) reported that no impact is likely, and 6% (5/87) did not know if it would have an impact.

## Discussion

### Quality of Access to Health Information

When asked about the quality of their current access to research articles, 65% (57/88) of participants in this study responded that it was good or excellent, while 32% (28/88) of respondents said it was poor or very poor. These responses need to be taken in light of the universal access among this sample to UpToDate. One survey respondent added that he receives complimentary journal subscriptions and, if he “had to pay, [he] wouldn't be reading them.”

The interview results yielded a similar picture of access to research, with six of the ten interviewees describing their quality of access as good or excellent, three as poor or very poor, and one as not applicable. Among those who felt that their access was inadequate, one hospital-based physician described the consequences of a low level of access to research:

H-P06 It’s a pretty regular basis where people are having problems [accessing articles]. If you can't pull up the articles, it's difficult to get all the information you need…it impacts our patient care.

A nurse at the hospital commented on the frequency of this issue, saying, “nine out of ten times, I can’t get all the articles that I want” [H-N09]. Current literature reports that perhaps a quarter of articles in PubMed are publicly accessible [20-22]. One might draw from this that potentially a third of the health personnel in this study, who feel their current access is inadequate, would be likely to welcome and take advantage of the NIH Public Access Policy.

### Source and Frequency of Access

In the survey, 83% (73/88) of respondents reported that they consult their colleagues on a weekly basis, which aligns with previous research [23]. Reference books and websites were utilized weekly by 69% (59/86) of the respondents. However, close to half (45%, 39/87) used UpToDate weekly. Additionally, the use of other POC resources such as MDConsult and Clinical Resources at Ovid suggest that the majority regularly relied on POC products. As previous studies have shown [24] and as our participant interviews affirmed, UpToDate is a preferred information source. A physician at the community clinic said:

C-P01 UpToDate is pretty much what most clinicians use when they have a clinical question. Most people just read the article, and they say, ‘This is what UpToDate says, so this is what I'm doing’.

She also described UpToDate’s appeal: “[It’s] simple. It's the iPod model” [C-P01]. A second physician at the clinic made similar observations but also discussed the limitations of UpToDate:

C-P02 It’s from one center....The protocols [are] very predigested….You know, it’s usually just written by a bunch of experts sitting in a room. Yeah, they have all the access to evidence, but you never know the sources of bias.

To our knowledge, few studies have assessed the quality of medical information in POC products such as UpToDate. Of those that do, one recent study compared the volume of content, editorial quality, and evidence-based methodology of 18 POC products [17]. UpToDate ranked in the top quartile for editorial quality and evidence-based methodology and the high intermediate quartile for the volume of content. However, another recent study that did an in-depth bibliometric analysis of five popular POC products (UpToDate, ACP PIER, Clinical Evidence, DynaMed, FirstCONSULT) found that three of the five products, including UpToDate, had nearly 50% or more citations from 2001 or earlier for the four topics analyzed (hypertension, asthma, carbon monoxide poisoning, and hyperlipidemia) [18]. The authors also found surprisingly little overlap in citations among the POC products for the four medical topics analyzed. While the authors acknowledge that this study only examined summaries of four topics, they conclude that it “reveals surprising and critical information about these POC products: they can vary greatly in content, from the raw number of citations, to the types of evidence, to the currency of those citations” [18]. They advise users to “judiciously appraise POC product information content” when using this information for EBP [18].

Our study suggests that fewer participants read review articles or research articles than turn to this POC. This finding must be considered in relation to the amount of current research available, the current difficulties in searching the biomedical literature, and how these factors will gradually improve over time. Given the potential limitations of “evidence” in POC products as well as in certain clinical guidelines [25-28], it may be necessary for health personnel to have access to the primary literature for the best evidence necessary for EBP.

In the interviews, a hospital physician described the frequency of his need for access: “It's pretty much on a day-to-day basis that you're looking for something” [H-P06]. A physician at the community clinic described her general information needs as “constant” [C-P01]. She attributed this to her natural curiosity but also to the complexity of patient issues and to the lack of easily accessible consultations from specialists.

C-P01 I have to look up stuff all the time, mainly because I'm curious about things, but also because people ask me questions, and I don't always know the answers. The level of questions…tend to be very sophisticated…because patients are so complex, [and] because our providers are really adept and are left to manage a lot of things that, in other places, specialists would be taking care of.

These finding are consistent with previous studies [9,11] that have found colleagues and reference textbooks to be physicians’ preferred information sources [6]. Several studies [29,30] have also cited research journals as “a primary mechanism for continuing medical education” [31]. Studies on nurses report a similar preference for local sources of information including patient and lab data as well as colleagues [32-35]. Nurses may also consult nursing journals if the content is relevant to the patient issue [11].

### Search Strategies

Although the majority of respondents turned to POC services, many also reached outside of these resources, turning to Google or Wikipedia more frequently than PubMed. This preference may be due to an interest in background information, which these resources provide, whereas research articles contain more specific foreground information. Studies have shown that 50% to 70% of physicians use Wikipedia as an information source [36]. Empirical studies have begun to emerge on the quality of Wikipedia’s medical content. These studies find few factual errors but also a general lack of depth and ease of understanding [37].

Ease of access and searching of these tools may contribute to their relative popularity. For example, a physician and administrator at the clinic said that she uses Google because it is “easy” and “in front of me” [C-P03]. She said that she typically sticks to “Google, UpToDate, or a consultant” because of this ease of access [C-P03].

Some health personnel used Google to access relevant research. As a physician at the clinic described: “In this setting, since you would have to pay for the articles…if you’re real interested in the answer to your question, you can just do some Google searches and shotgun around and see if some institutional setting has an unprotected link to it” [C-P02]. Similarly, a nurse at the hospital said that she always starts with Google but is careful to “always look for websites that have .edu or. org or .gov” as opposed to “commercial websites” [H-N09].

PubMed or MEDLINE was the fourth most frequently reported resource, reported to be used on a weekly basis by 27% (24/89) of health personnel. The interview participants elaborated on the various uses of PubMed. A physician at the clinic said that one of her “most common PubMed searches,” which she conducts approximately weekly, is searching for “a review article from core clinical journals within the last five years” [C-P01]. A hospital physician said he uses PubMed “to educate other people and myself,” to prepare for talks and for rare patient cases, such as “an unusual hemoglobinopathy” (H-P10). He added that he typically uses PubMed “in retrospect” because “it takes a while if you're researching articles to find something” [H-P10].

Other participants described their PubMed strategies and frustrations. A hospital nurse described how she typically accesses PubMed via Google: “I just enter what I want to know on Google…and if I see PubMed, I always go to that because I trust it as a source of the latest research” [H-N09]. She added, however, that she has “not had much success navigating [PubMed]” [H-N09]. A medical fellow at the hospital described how he “used to use PubMed” but switched to Google Scholar because it seemed “more user friendly,” “pulled things up better,” and generally “works better” [H-P06]. A hospital nurse added, “I primarily use [PubMed] if I know the exact citation” [H-N05]. Training in PubMed has been shown to foster favorable information-seeking behavior, such as increased searches in MEDLINE [38]. Our findings suggest that training in PubMed may bolster the impact of the NIH Public Access Policy.

Comparisons of preappraised information sources with the primary literature have shown, for example, that preappraised sources could not provide answers to 40% of the complex clinical questions, 95% of which had previously been answered by the primary research literature [13]. Other studies have similarly pointed to the value of access to the research literature in addition to POCs [14,39].

Only 9% (8/87) of survey respondents had access to a medical school library’s online resources. This affiliation provides the user with remote access via a secure user name and password to the library’s online full-text journal subscriptions. It should be noted that individuals with these privileges acknowledge upon receipt of their access that they will not share this information, as sharing is a violation of the library’s contract with publishers. Related to the value of a password, a physician at the community clinic noted that remote access is one of the major benefits of training residents: “You either don’t get paid, or don’t get paid much, but you *do* get access to that library” [C-P02]. Several interviewees described how individuals with passwords to the medical school library are often asked to retrieve articles online for others. A hospital nurse, who frequently used this method of access, said:

H-N09 I never [pay for articles]. I just call different friends that have access to different services and do what I need to do.

A hospital physician and clinical professor described how “people will ask [him] to get articles through the library” as often as “a couple times a month” [H-P10]. He concluded, “My assumption is that they’re searching, and for whatever reason, it’s not an open-access article, and so they’re asking me to get it from [the university library]” [H-P10]. A medical fellow at the hospital said that he has been using his friend’s password to access the university library for a number of years. When asked whether he could remember any instances of having difficulty accessing a particular article, he said:

H-P06 I've been cheating the system for so long that I don't remember....Pretty much every single article is on their site.

### Reasons for Access

In the current study, the most popular reason for consulting research on a weekly basis was for “informing my understanding of a specific patient” (35%, 31/88), a finding that has been demonstrated in previous research [40]. Several interviewees related stories of patient care that hinged on access to research articles. For example, a medical fellow at the hospital described how access to research was necessary in treating a complex case:

H-P06 There was a kid who had hemorrhagic cystitis…and we were trying to figure out an appropriate way to treat that, and it's a rare, complicated thing. And so having access to the articles gave potential treatment options, which, in this case, was actually injecting the bladder with an agent that helps you to clot. Within a day, we got the articles that we needed, and we were able to start that treatment, which helped the kid.

In this case, the medical fellow used his friend’s password to a university library to gain remote access to the necessary articles. He said that the full-text articles were necessary because with abstracts only “you're not going to get enough information to make a meaningful decision” (H-P06). A community clinic physician described a similar scenario involving patient treatment, in which he was unable to freely access the article that he needed:

C-P02 A question came up a little while ago about what oral antibiotics you can take for bone infections in the outpatient setting…it made the difference between the patient getting six months of IV antibiotics at home…or just getting six weeks’ oral antibiotics without needing a intravenous capillary…and I ended up paying 50 bucks for the article.

The second most frequently reported reason for accessing research on a weekly basis was out of general interest (31%, 26/84). As a clinic physician said, “At any given moment, I'll have two folders full of things that I'm reading” [C-P01]. Similarly, a nurse at the hospital said that she reads anything that interests her: “It kind of intrigues [me] - I’m naturally curious - to read about head lice, which has no relevance to anything I do clinically, but I think it's interesting” [H-N05].

Studies have also demonstrated the association between physician use of an online evidence system with patient admissions, suggesting that evidence use was related to patient care [41]. Additional research is needed to empirically test whether access itself would lead to increased use of research literature. A physician at the community clinic nicely summarized the issue at hand:

C-P02 I view [reading research] as necessary, but something that I do very little, just to be completely honest…. You just learn that you don’t have that many resources available to you, or, if you do, it’s a pain to get them at the point of care. So it’s the question of the chicken or the egg. Do we not do it because we’re not that interested? Or do we not do it because it’s such a pain to find it, and we become accustomed to not doing it?

The physician raises an important empirical question that warrants further study. He seemed to be describing a sort of *learned helplessness* that occurs when the needed information is not available. Future research under consideration, given the warrant provided by the results of this study, is a randomized controlled trial (RCT) comparing the uses of research by a *high-access group* that receives relatively complete access to the biomedical research literature and another *same-access group* with typical (unchanged) access. A third group, a *high-access group with support from medical librarians*, could also be included in order to examine the value-added benefit of training and support. Ideally, this research would be longitudinal, as few studies examine health personnel use of online evidence over an extended period of time [42].

### Limitations of the Study

While encouraging, our study has limitations. For example, participation for health personnel was only solicited from two health care sites, which means that these findings may or may not be applicable outside of these two institutions. Also, this study only analyzed self-reported behaviors and preferences, which may differ from actual practices. As Covell [9] has demonstrated, physicians tend to overestimate their actual use of printed information resources, while underestimating their use of peer consultation. Lastly, a relatively small number of health personnel were surveyed and, as with many voluntary surveys, our sample may be biased. Future plans to increase sample size and the number of participants will be considered for additional research.

Finally, future research may benefit from examining the potential impact of the NIH policy among other participant groups, such as patients or researchers. Since many individuals are seeking health information online - attracted to the convenience, coverage, and anonymity of online health information [43] - it may be beneficial to track the potential impact of the NIH policy within this population. Similarly, the NIH Public Access Policy is expected to be beneficial for biomedical researchers without access to well-funded research libraries or who do not work in one of the hundred-plus of the world’s poorest countries that qualify for the Health Access to Research program (HINARI) [44]. Of course, a recent RCT demonstrated that articles assigned to the open access condition received more downloads than control articles, and the authors concluded that the true beneficiaries of open access publishing may be consumers, not producers, of the medical literature [45], such as the health personnel studied here.

### Conclusions

This study establishes a preliminary measure of current research use, interest, and barriers among a sample of health personnel in hospital and community clinic contexts. While health personnel have limited time available for consulting additional sources and are already equipped with POC services, the results still provide grounds for expecting the NIH Public Access Policy to have a positive impact on EBP and health care more generally, given that between a quarter and a third of the participants (1) frequently access research literature, (2) express an interest in having greater access, and (3) are aware of the NIH policy and expect it to have an impact on their accessing the research literature. Additional measures are warranted if health personnel and their patients are going to maximize the benefits from this increased access to research through policy promotion, medical education, continuing website improvements to PubMed, and research on the nature of clinical practices and decision making in light of this increased access to the research literature.

## Abbreviations

EBP: evidence-based practice
POC: point of care
RCT: randomized controlled trial
